# Elevated lactate dehydrogenase predicts pneumonia in spontaneous intracerebral hemorrhage

**DOI:** 10.1016/j.heliyon.2024.e26109

**Published:** 2024-02-13

**Authors:** Yangchun Xiao, Shuanghong He, Xin Cheng, Liyuan Peng, Yixin Tian, Tiangui Li, Jialing He, Pengfei Hao, Weelic Chong, Yang Hai, Chao You, Fang Fang, Zongjun Peng, Yu Zhang

**Affiliations:** a Department of Neurosurgery, Clinical Medical College and Affiliated Hospital of Chengdu University, Chengdu, Sichuan, China; b Health Management Center, West China Hospital, Sichuan University, Chengdu, Sichuan, China; c Department of Neurosurgery, West China Hospital, Sichuan University, Chengdu, Sichuan, China; d Department of Neurosurgery, The First People's Hospital of Longquanyi District Chengdu, Sichuan, China; e Department of Neurosurgery, The Second Affiliated Hospital of Guangzhou Medical University, Guangzhou, Guangdong, China; f Department of Neurosurgery, Shanxi Provincial People's Hospital, Taiyuan, Shanxi, China; g Department of Medical Oncology, Thomas Jefferson University, Philadelphia, PA, USA; h Sidney Kimmel Medical College, Thomas Jefferson University, Philadelphia, PA, USA; i Department of Neurosurgery, Sichuan Friendship Hospital, China

**Keywords:** Intracerebral hemorrhage, Lactate dehydrogenase, Pneumonia, Complication, Inflammatory marker

## Abstract

**Background:**

Although a variety of risk factors for pneumonia after spontaneous intracerebral hemorrhage have been established, an objective and easily obtainable predictor is still needed. Lactate dehydrogenase is a nonspecific inflammatory biomarker. In this study, we aimed to assess the association between lactate dehydrogenase and pneumonia in spontaneous intracerebral hemorrhage patients.

**Methods:**

Our study was a retrospective, multicenter cohort study, undertaken in 7562 patients diagnosed with spontaneous intracerebral hemorrhage from 3 hospitals. All serum Lactate dehydrogenase was collected within 7 days from admission and divided into four groups as quartile(Q). We conducted a multivariable logistic regression analysis to assess the association of Lactate dehydrogenase with pneumonia.

**Results:**

Among a total of 7562 patients, 2971 (39.3%) patients were diagnosed with pneumonia. All grades of elevated lactate dehydrogenase were associated with increased raw and risk-adjusted risk of pneumonia. Multiple logistic regression analysis showed odds ratios for Q2-Q4 compared with Q1 were 1.21 (95% CI, 1.04–1.42), 1.64(95% CI, 1.41–1.92), and 1.92 (95% CI, 1.63–2.25) respectively. The odds ratio after adjustment was 4.42 (95% CI, 2.94–6.64) when lactate dehydrogenase was a continuous variable after log-transformed.

**Conclusions:**

Elevated lactate dehydrogenase is significantly associated with an increase in the odds of pneumonia and has a predictive value for severe pneumonia in patients with pneumonia. Lactate dehydrogenase may be used to predict pneumonia events in spontaneous intracerebral hemorrhage patients as a laboratory marker.

## 1 Introduction

Spontaneous intracerebral hemorrhage (ICH) is a grave condition affecting approximately 3.14 million patients worldwide, with 485,474 patients in China alone [[1], [2], [3], [4]]. ICH carries substantial mortality and disability rates, imposing a significant burden on families and society. Over the past decades, there has been a significant increase in the number of ICH cases and related deaths [[1], [2], [3]], highlighting the urgent need to address this major public health concern. Pneumonia is a major complication after ICH, and is associated with a higher rate of worse outcomes in ICH patients [[5], [6], [7]]. The incidence rate of pneumonia in ICH patients continues to be high about 14.3–31.1% [8,9]. Several clinical trials evaluated the use of antibiotics to prevent pneumonia after stroke; However, the outcomes of these trials prove that prophylaxis antibiotic is ineffective to prevent pneumonia [[10], [11], [12], [13]]. Thus, a new and effective predictor is needed to identify ICH patients at high risk of pneumonia and guide early antibiotic use.

In prior studies, several risk factors have been identified to predict pneumonia, including older age, current smoking, admission Glasgow Coma Scale (GCS), National Institutes of Health Stroke Scale score, chronic obstructive pulmonary disease, and dysphagia. Laboratory biomarkers have gained significant attention because of their practicality, sensitivity, and convenience. However, the majority of biomarkers necessitate specialized laboratory tests such as C-reactive protein (CRP) [14] and interleukin-6 (IL-6) [15], which can lead to added expenses. Thus, more reliable and accessible measurable blood markers needed to be found for early prognostication of pneumonia and guiding early antibiotic use.

Lactate dehydrogenase (LDH) is a well-known serum marker of inflammation and infection [16,17]. LDH showed a good ability to predict bacterial pneumonia, tuberculosis, pneumocystis carinii pneumonia, and as surrogate markers for steroid therapy for Mycoplasma pneumoniae pneumonia in prior studies [[18], [19], [20], [21], [22]]. However, no studies have been conducted to evaluate the predictive ability of LDH for pneumonia in patients with ICH. Therefore, the objective of this multicenter cohort study was to assess the association between LDH, easily obtained through laboratory tests, and pneumonia in ICH patients. Our study aims to contribute to the existing literature on this topic.

## 2 Methods

### 2.1 Study design

Our study was a retrospective, multicenter cohort study. This study was taken on 7562 patients diagnosed with ICH who either underwent pneumonia or did not during hospitalization between December 2010 and August 2019 from West China Hospital Sichuan University, between December 2016 and November 2020 from The First People's Hospital of Longquanyi District Chengdu, and between August 2012 and November 2020 from Affiliated Hospital of Chengdu University. This study focused on the association between LDH and the occurrence of pneumonia during hospitalization. The study was approved by the institutional review boards of the ethics committees of three hospitals: West China Hospital (No. 2021-624), The First People's Hospital of Longquanyi District Chengdu (No. AF-AK-2022010), and The Affiliated Hospital of Chengdu University (No. PJ2021-017-03). As it involved a clinical audit, informed consent was waived. The study complied with the STROBE criteria and followed the ethical principles of the Declaration of Helsinki 1964.

### 2.2 Patient selection

All patients who were diagnosed with spontaneous ICH were taken in. All ICH patients were confirmed at admission through computed tomography or magnetic resonance imaging and intraoperative diagnosis by a neurosurgeon during hospitalization. All the patients were over 18 years.

Patients with the following criteria were excluded [1]: ischemic stroke with hemorrhagic transformation, trauma, cerebral aneurysm, arteriovenous malformations, or hemorrhagic disorders due to coagulation abnormalities, and other diseases are different from primary ICH [2]; infections or community-acquired pneumonia before admission [3]; patients whose serum LDH were not obtained within 24 h from admission.

Clinical Characteristics and Laboratory Data Collection.

We collected the following demographic characteristics and clinical features including age, gender, current smoking status, alcohol consumption, hypertension, diabetes mellitus, hematoma location, hematoma volume, and intraventricular hematoma. We also assessed ICH severity at admission using GCS and treatment. General laboratory tests including LDH, leukocyte, lymphocyte, albumin, glucose, and platelets were conducted within 7 days from admission. We divided patients into four groups based on serum LDH levels as quartiles.

### 2.3 Exposure

In our study, the primary exposure variable was the serum LDH level upon admission. All serum LDH samples for admission were the first time collected within 24 h of admission. Furthermore, LDH levels were measured during the entire hospital stay, with a maximum duration of 10 days. We also collected the median and maximum LDH levels within 7 days after admission. According to the previous studies [23,24], the patients were grouped into quartiles based on their LDH levels. This grouping enabled us to understand the correlation between LDH levels and pneumonia risk and to determine if a dose-response relationship exists. The patients were categorized into four groups based on LDH levels: Q1 (<163 U/L), Q2 (163–192 U/L), Q3 (193–231 U/L), and Q4 (>232 U/L).

### 2.4 Outcomes

The primary outcome was pneumonia in ICH patients. Pneumonia was defined as lower respiratory tract infections that satisfied the Modified Centers for Disease Control and Prevention criteria, which also incorporated diagnostic alterations observed on at least one chest x-ray or chest computed tomography [25].

We assess the severity of pneumonia with the pneumonia severity index (PSI) and CURB-65 score system (confusion, urea, respiratory rate, blood pressure, and age over 65 years) [26,27]. PSI was based on age, coexisting disease, abnormal physical findings, and abnormal laboratory findings. CURB-65 score system depends on confusion, urea>7 mmol/L, respiratory rate ≥30, systolic blood pressure≤ 90 or diastolic blood pressure≤ 60, and age over 65 years. According to the PSI score, patients were divided into a low-risk group (PSI ≤90) and a high-risk group (PSI>90). According to the CURB-65 score, patients were also divided into a low-risk group (CURB-65 1–2 scores) and a high-risk group (CURB-65 3–5 scores).

### 2.5 Statistical analysis

Continuous data including age, GCS, hematoma volume, leukocyte, lymphocyte, albumin, platelets, and glucose were shown as means and standard deviations. We compared continuous variables with the Student's t-test and the Mann-Whitney *U* test. The categorical data were analyzed by calculating frequencies or percentages and compared with the chi-square test or Fisher's exact test. We assessed the relationship between the LDH and pneumonia severity in different PSI groups and CURB-65 groups. A two-sided P value < 0.05 was regarded as statistically significant. We used median imputation to handle missing data.

All available demographics, baseline variables, and laboratory tests were selected as factors in logistic regression models according to prior studies and clinical expertise. Univariate logistic regression models were separately conducted to analyze the association between factors and outcomes. Factors (P value < 0.10) were selected as confounders for the multivariable logistic regression analysis. In multivariable logistic regression analysis.

Restricted cube spline was used to assess the odds of risk of pneumonia with LDH after log transformation. A trajectory chart was used to show the LDH levels over time among all patients. Furthermore, we also conducted a dose-response analysis to evaluate the effect between LDH and the rate of pneumonia.

We used the receiver operating characteristic (ROC) curve and the area under the receiver operating characteristic curve (AUROC) to calculate leukocyte, lymphocyte, albumin, glucose, and platelets in predicting pneumonia. AUROC also be used to evaluate the discrimination of admission LDH, maximum LDH, and median LDH in predicting pneumonia. Furthermore, we also compared prediction models with AUROC of LDH per day after admission.

Further subgroup analyses were conducted based on age (≤65 years and >65 years), sex (male and female), smoking, alcohol, hypertension, diabetes, hematoma location (infratentorial and supratentorial), hematoma volume (≤30 ml and >30 ml), intraventricular hematoma, GCS (<9 scores and ≥9 scores), clearance of intracranial hematoma to assess the heterogeneity of outcomes. Admission LDH was divided into low groups and high groups based on the median value of LDH (193 U/L). All the factors were selected based on clinical advice, prior studies, and a review of risk scores. All statistical analyses were conducted with R software (version 4.2.1; Foundation for Statistical Computing). A P-value less than 0.05 was regarded as significant. All p values were two-sided.

## 3 Results

A total of 6348 patients from West China Hospital, 780 patients from The First People's Hospital of Longquanyi District Chengdu, and 1935 patients from The Affiliated Hospital of Chengdu University, who were diagnosed with ICH and were over 18 years old, were identified. Patients who had conditions other than primary ICH (877 patients) and those without LDH level measurements at admission (624 patients) were excluded. Consequently, our study included a total of 7562 patients diagnosed with ICH from the three hospitals (as shown in eFig. 1). Among them, 2971 patients (39.3%) had pneumonia. The baseline characteristics between groups are shown in Table 1. We found that patients with higher LDH levels were older, less female, more smoking, more often with diabetes, and appeared to arise with bigger hematoma volume and lower GCS score, more often with intraventricular hematoma, higher leukocytes, higher neutrocyte, lower lymphocyte, and lower albumin.

**Fig. 1:**
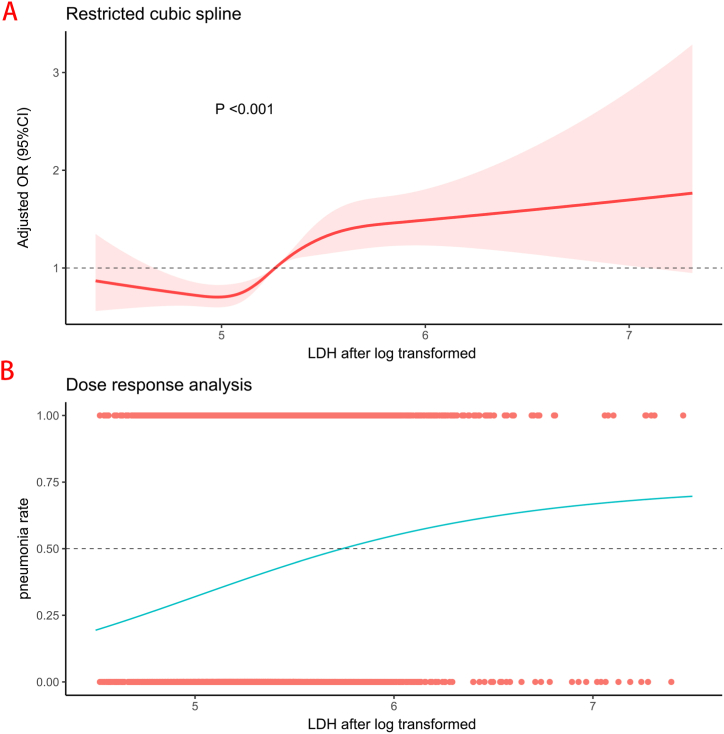
Adjusted Odds Ratio (OR) and 95% Confidence Interval (CI) Are Shown for Each 20 U/L Change Away from The Reference Value (100 U/L). High Admission Lactate Dehydrogenase Was Associated with a Higher Proportion of Pneumonia. A: The figure shows an apparent trend of adjusted OR of LDH with pneumonia. B: The figure shows an apparent dose-response trend in pneumonia incidence associated with LDH levels. The points show patients with pneumonia (y-axis = 1) and without pneumonia (y-axis = 0). All the LDH log transformed is a log with base e. LDH: Lactate Dehydrogenase.

**Table 1:** Baseline characteristics of spontaneous intracerebral hemorrhage patients stratified by baseline admission lactate dehydrogenase levels.

Characteristics	Lactate Dehydrogenase (U/L)				
	Q1<163 (n = 1933)	Q2 163–192 (n = 1902)	Q3 193–231 (n = 1859)	Q4 ≥232 (n = 1868)	P value
Demographics					
Age, year, mean (SD)	55.56 (15.09)	59.14 (14.37)	59.59 (14.48)	59.32 (14.82)	<0.001
Female, n (%)	544 (28.1)	609 (32.0)	640 (34.4)	719 (38.5)	<0.001
Current Smoking, n (%)					0.015
Never	1233 (63.8)	1282 (67.4)	1269 (68.3)	1283 (68.7)	
Ever	123 (6.4)	126 (6.6)	103 (5.5)	99 (5.3)	
Current	577 (29.8)	494 (26.0)	487 (26.2)	486 (26.0)	
Alcohol abuse, n (%)	669 (34.6)	588 (30.9)	574 (30.9)	589 (31.5)	0.039
Medical history, n (%)					
Hypertension	1198 (62.0)	1304 (68.6)	1273 (68.5)	1140 (61.0)	<0.001
Diabetes	210 (10.9)	188 (9.9)	195 (10.5)	169 (9.0)	0.27
Hematoma characteristics					
Supratentorial hematoma, n (%)	356 (18.4)	358 (18.8)	323 (17.4)	388 (20.8)	0.06
Size of hematoma, cm, mean (SD)	19.26 (23.46)	21.01 (28.88)	21.57 (24.74)	28.31 (33.86)	<0.001
Intraventricular hematoma, n (%)	353 (18.3)	421 (22.1)	509 (27.4)	628 (33.6)	<0.001
Glasgow coma score, mean (SD)	12.24 (3.54)	11.64 (3.80)	10.96 (3.97)	9.10 (4.36)	<0.001
Operation					
Hematoma clearance, n (%)	554 (28.7)	456 (24.0)	417 (22.4)	377 (20.2)	<0.001
Laboratory tests					
Leukocyte, 10 9/L, mean (SD)	7.62 (3.02)	8.34 (3.44)	9.14 (3.89)	11.81 (18.31)	<0.001
neutrocyte, 10 9/L, mean (SD)	15.77 (24.71)	15.52 (24.41)	18.39 (26.57)	23.57 (30.18)	<0.001
lymphocyte, 10 9/L, mean (SD)	1.07 (0.60)	1.01 (0.61)	0.98 (0.70)	1.06 (1.03)	<0.001
Albumin, mg/L, mean (SD)	36.35 (6.07)	37.29 (5.78)	37.37 (6.20)	36.99 (7.14)	<0.001
Platelets, 10 9/L, mean (SD)	153.70 (64.43)	151.73 (64.02)	151.01 (65.73)	151.49 (86.59)	0.67
glucose, mmol/L, mean (SD)	5.77 (2.50)	6.25 (2.70)	6.66 (3.21)	7.49 (4.18)	<0.001

The primary outcome results of univariate logistic regression and adjusted multivariable analyses are shown in Table 2. The odd ratios (ORs) compared with Q1 LDH levels were 1.14 (95% CI, 1.00–1.31) for Q2, 1.62 (1.42–1.85) for Q3, and 2.28 (95% CI, 2.00–2.60) for Q4 analyzed by univariate logistic regression. The ORs after adjusted potential confounders by multivariable logistic regression analysis remained significant were 1.21 (95% CI, 1.04–1.42) for Q2, 1.64 (95% CI, 1.41–1.92) for Q3, and 1.92 (95% CI, 1.63–2.25) for Q4. Factors selected in the multivariable logistic regression analysis were age, sex, smoking, alcohol abuse, diabetes, size of the hematoma, hematoma clearance operation, size of the hematoma, intraventricular hematoma, GCS, leukocyte, lymphocyte, albumin, and platelets (shown in eTable 1). The adjusted OR was 4.42 (95% CI, 2.94–6.64) when LDH were log-transformed and analyzed as continuous. The odds ratio between the LDH and In-hospital mortality were 1.26 (95% CI, 0.90–1.76) for Q2, 1.41 (95% CI, 1.02–1.95) for Q3, and 1.90 (95% CI, 1.40–2.58) for Q4 (shown in eTable 2). The odds ratio between the log-transformed delta LDH (calculated by subtracting the admission LDH level from the LDH level one week after admission) and the occurrence of pneumonia was 1.28 (95% CI, 1.09–1.49).

**Table 2:** Unadjusted and adjusted associations between pneumonia Amount admission, median, and maximum lactate dehydrogenase levels.

Lactate dehydrogenase (U/L)		Events, n (%)	Unadjusted OR	P trend	Multivariable Regression adjusted OR	P trend
Admission	Log transformed	2971/7562 (39.3%)	6.83 (4.85–9.60)	<0.001	4.42 (2.94–6.64)	<0.001
	Q1 <163	599/1933 (31%)	1 [Reference]		1 [Reference]	
	Q2 163-192	645/1902 (33.9%)	1.14 (1.00–1.31)	0.05	1.21 (1.04–1.42)	0.02
	Q3 193-231	782/1859 (42.1%)	1.62 (1.42–1.85)	<0.001	1.64 (1.41–1.92)	<0.001
	Q4 ≥232	945/1868 (50.6%)	2.28 (2.00–2.60)	<0.001	1.92 (1.63–2.25)	<0.001
Median	Log transformed	2971/7562 (39.3%)	11.12 (7.86–15.73)	<0.001	3.89 (2.59–5.84)	<0.001
	Q1 <176	505/1919 (26.3%)	1 [Reference]		1 [Reference]	
	Q2 176-207	673/1895 (35.5%)	1.54 (1.34–1.77)	<0.001	1.47 (1.25–1.72)	<0.001
	Q3 208-250	833/1858 (44.8%)	2.28 (1.98–2.61)	<0.001	1.84 (1.57–2.16)	<0.001
	Q4 ≥251	960/1890 (50.8%)	2.89 (2.52–3.31)	<0.001	1.98 (1.68–2.34)	<0.001
maximum	Log transformed	2971/7562 (39.3%)	26.27 (19.16–36.04)	<0.001	4.85 (3.37–6.98)	<0.001
	Q1 <189	412/1934 (21.3%)	1 [Reference]		1 [Reference]	
	Q2 189-229	612/1859 (32.9%)	1.81 (1.57–2.10)	<0.001	1.56 (1.32–1.84)	<0.001
	Q3 230-293	880/1899 (46.3%)	3.19 (2.77–3.67)	<0.001	2.15 (1.83–2.54)	<0.001
	Q4 ≥294	1067/1870 (57.1%)	4.91 (4.26–5.66)	<0.001	2.41 (2.03–2.86)	<0.001

Multivariable Regression adjusted by age, sex, smoking, alcohol abuse, diabetes, size of the hematoma, hematoma clearance operation, size of the hematoma, intraventricular hematoma, Glasgow Coma Scale score, leukocyte, lymphocyte, albumin, and platelets.

A continuous relationship between admission LDH levels and pneumonia were shown in Fig. 1. We found that the association between admission LDH levels and pneumonia was strong (P = 0.004). The ORs of pneumonia significantly elevated with LDH levels. The dose-response association was shown in Fig. 1. We found that elevated LDH was associated with an increased rate of pneumonia.

We assessed the admission LDH levels between the low-risk PSI group and high-risk PSI group in pneumonia patients (as shown in eFig. 2), There was a significant difference between the two groups, the P value was 0.004. We also observed a significant difference in different CURB-65 groups in pneumonia patients, the P value was less than 0.001.

**Fig. 2:**
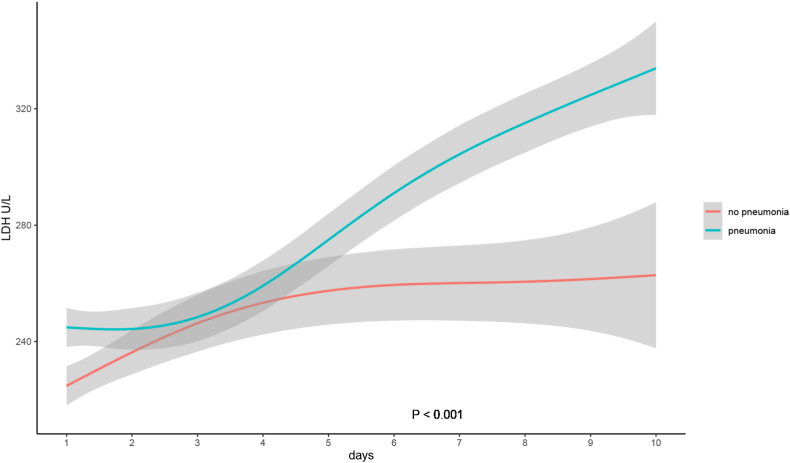
Lactate dehydrogenase levels in 10 Days after admission over time among patients with Pneumonia LDH: Lactate dehydrogenase.

The trajectory chart showed the LDH levels over time among patients with pneumonia or not in Fig. 2. We observed that The LDH levels were continuously elevated over time. LDH levels of pneumonia patients were higher compared to patients without pneumonia, the P value was statistically significant <0.001.

**Fig. 3:**
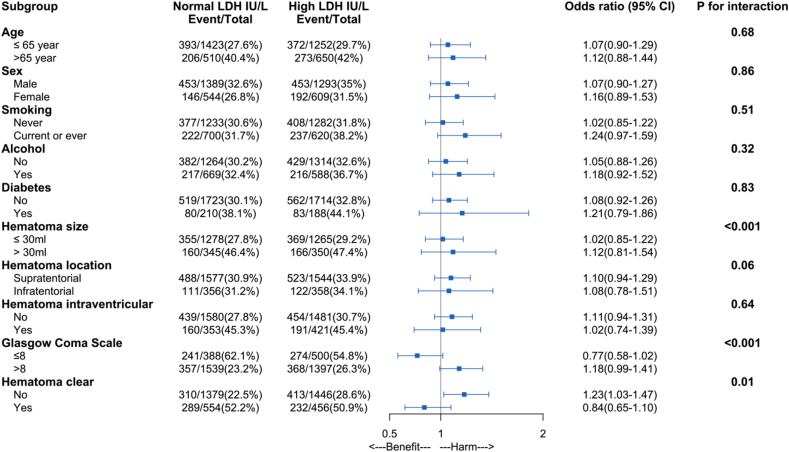
The association of admission Lactate Dehydrogenase and pneumonia among patients with Spontaneous intracerebral hemorrhage, stratified by various demographic and clinical characteristics. LDH: Lactate Dehydrogenase.

All subgroup analysis results were reported in Fig. 3. We observed significant interaction in GCS<9 groups and GCS ≥9 group (P < 0.001). Patients with higher LDH levels were associated with a high risk of pneumonia compared to low levels in GCS ≥9 groups (1.18 (95% CI, 0.99–1.41)). Patients in GCS≤ 8 groups showed that higher LDH levels were associated with less risk of pneumonia (0.77 (95% CI, 0.58–1.02)). The other significant interaction was observed in the hematoma volume subgroups (≤30 ml versus >30 ml) (1.02 (95% CI, 0.85–1.22) versus 1.12 (95% CI, 0.81–1.54), P < 0.001). No significant interactions between LDH and other interesting factors in pneumonia were observed.

**Fig. 4:**
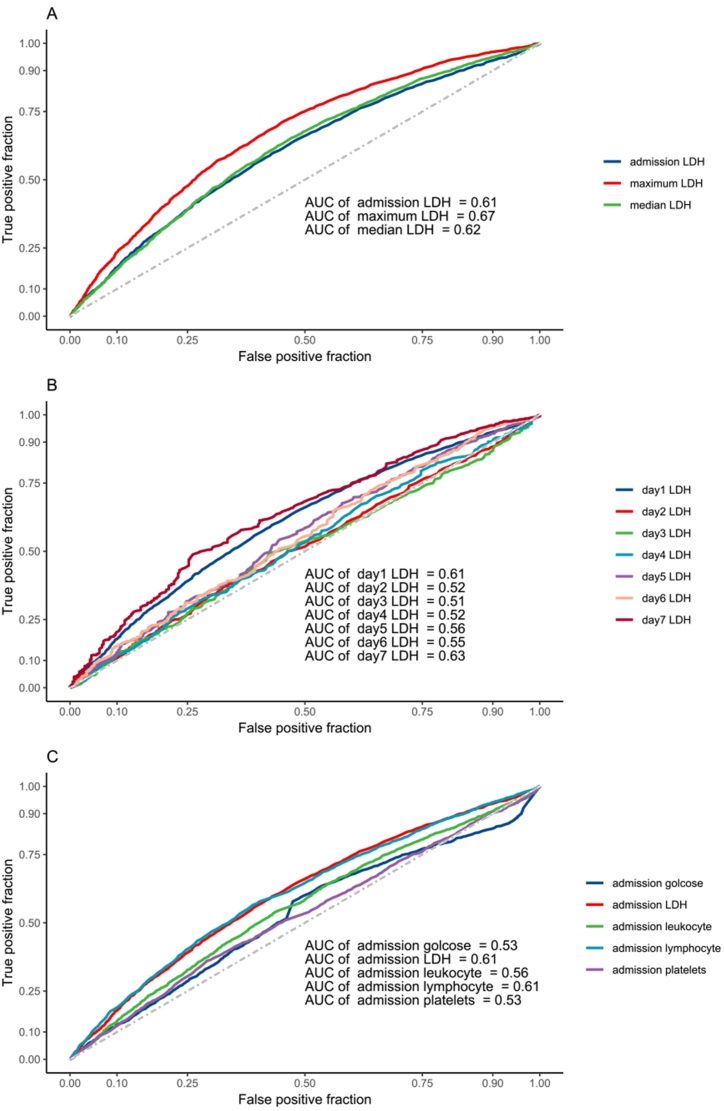
Receiver operating characteristic curves of Lactate Dehydrogenase and other laboratory marks for pneumonia. Comparison of area under the receiver operating characteristic curve values among admission LDH, median LDH, and maximum LDH after admission within 7 days. Comparison of area under the receiver operating characteristic curve values among LDH per day after admission within 7 days. Comparison of area under the receiver operating characteristic curve values among LDH, glucose, leukocyte, lymphocyte, albumin, and platelets at admission. LDH: Lactate dehydrogenase AUC: area under the receiver operating characteristic curve.

ROC analyses were shown in Fig. 4. The AUROC was produced to compare the ability of LDH levels in the prediction of pneumonia. The AUROC was 0.61 for the admission LDH levels, 0.67 for max LDH levels, and 0.62 for median LDH levels. We continually compared AUROC of everyday LDH levels within 6 days after admission, 0.61 for the first day, 0.52 for the second day, 0.51 for the third day, 0.52 for the fourth day, 0.56 for the fifth day, 0.55 for the sixth day, and 0.63 for the seventh day. We also used AUROC of other inflammatory markers to predict the incidence of pneumonia, 0.61 for admission LDH levels, 0.53 for glucose, 0.56 for leukocytes, 0.61 for lymphocytes, 0.67 for albumin, and 0.53 for platelets.

## 4 Discussion

In this large study, we found that higher LDH levels were strongly associated with pneumonia in ICH patients. Furthermore, Higher admission LDH levels were significantly associated with more pneumonia severity (PSI >90 and CURB-65 scores ≥3) in ICH patients with pneumonia. Additionally, our study demonstrated a significant correlation between higher admission LDH levels (>193 U/L) and in-hospital mortality.

In previous studies, LDH was reported to predict early hematoma expansion and poor outcomes in patients with ICH [28,29]. Yan et al. reported that a higher LDH to albumin ratio was associated with the risk of stroke-associated pneumonia in patients with acute ischemic stroke [30]. Ding et al. found that elevated LDH levels predicted postoperative pneumonia after surgery in patients with aneurysmal subarachnoid hemorrhage [31]. Our study was the first study to assess the association between LDH levels and pneumonia in patients with ICH.

Several potential explanations for the association between LDH levels and pneumonia have been listed. Firstly, LDH is a widely recognized enzyme distributed in various body tissues, including the kidneys, heart, liver, and lungs. Pneumonia can result in direct injury to lung tissue, resulting in the release of LDH into the bloodstream [32]. Secondly, LDH is associated with excessive inflammation of pulmonary tissue [32,33]. LDH levels have been utilized to evaluate the severity of community-acquired infections caused by Mycoplasma pneumoniae and coronavirus disease 2019 [[18], [19], [20]]. Finally, LDH plays a vital role in the metabolism of pneumococcal pyruvate, as well as the survival of pneumococcus in the bloodstream. Moreover, it may also contribute to enhancing the virulence of Streptococcus pneumoniae [34,35].

LDH can serve as an advantageous predictor of pneumonia due to its cost-effectiveness and high sensitivity and specificity. LDH testing is relatively simple, widely available, and economically feasible. This makes it a practical option for screening and monitoring pneumonia compared to complex or expensive biomarkers, such as CRP, IL-6, and TNF-a. Its lower cost allows for increased accessibility in resource-constrained healthcare settings. Furthermore, previous studies have shown that LDH performs comparably to other biomarkers in predicting pneumonia [36,37]. This study has found that elevated LDH levels in patients with ICH were associated with a higher risk of developing pneumonia. Its ability to accurately identify pneumonia helps in early diagnosis and appropriate management. However, this study did not compare LDH with certain biomarkers of inflammation, such as CRP, IL-6, and TNF-a, as they are not routinely assessed upon admission in clinical practice. Instead, we compared it to leukocyte count and found that LDH exhibited a superior predictive value in comparison.

Currently, there is limited research specifically addressing the effect of lowering LDH levels on pneumonia. In our study, the log-transformed delta LDH showed potential statistical significance, with an odds ratio of 1.28 (95% CI, 1.09–1.49). The results demonstrated an association between lowering LDH and the reduction of pneumonia in patients with ICH. Subsequent research should investigate potential therapeutic interventions targeting the reduction of LDH levels and their impact on pneumonia outcomes.

Some strengths of our analysis are listed. First, the sample size of our study was large, with 7562 patients from three independent hospitals. Second, we also correlated LDH with the date after admission and compared the AUROC of LDH per day after admission and compared the AUROC of max LDH, median LDH, and admission LDH to predict the incidence of pneumonia. Third, we compared the AUROC of LDH with other inflammatory markers to predict the incidence of pneumonia.

Our analysis has some limitations. First, this was a retrospective study. Although our study contains a large sample size, there was still a possibility of selection bias. Some patients discharged early may generate bias. Second, although all patients diagnosed with pneumonia were checked by an infectious physician, our study cannot accurately exclude all patients with active infections before admission. Third, all the data of LDH, chest computed tomography, and chest X-ray were obtained from the electronic medical record. We could not exclude bias from misreporting. Last, it is important to note that the quality of ICH care plays a significant role in determining pneumonia outcomes [38]. Regrettably, our research is limited by the absence of data on ICH care, potentially introducing bias to our findings.

## 5 Conclusion

Elevated admission LDH is significantly associated with an increase in the odds of pneumonia and has a predictive value for severe pneumonia in patients with pneumonia. LDH levels could be used to predict pneumonia events in ICH patients and guide antibiotic use. LDH is economical and could be easily obtained during hospitalization. Furthermore, prospective studies are needed to confirm the exact relationship between LDH and pneumonia.

## Financial disclosure statement

None.

## Ethical approval and consent to participate

The ethics committee of West China Hospital (No. 2021-624). The ethics committee of Affiliated Hospital of Chengdu University (No. PJ2021-017-03). The ethics committee of The First People's Hospital of Longquanyi District Chengdu (No.AF-AK-2022010). The committee waived the requirement for informed consent because the data were analyzed anonymously.

## Consent for publication

Not applicable.

## Availability of data and materials

All data generated or analyzed during this study are included in this article. Further inquiries can be directed to the corresponding author.

## Funding

This work is supported by National Natural Science Foundation of China (82271364), the innovation team project of Affiliated Hospital of Clinical Medicine College of Chengdu University (CDFYCX202203), and the project of Sichuan Science and Technology Bureau (22ZDYF0798), the 1·3·5 project for disciplines of excellence-Clinical Research Incubation Project, West China Hospital, Sichuan University (21HXFH046), the project of health commission of Sichuan province (2019HR50), Nursing Association of Sichuan Province H21003, and Science and Technology Research Project of Chongqing Municipal Education Commission.

## CRediT authorship contribution statement

**Yangchun Xiao:** Writing – original draft, Methodology, Formal analysis, Data curation. **Shuanghong He:** Formal analysis, Data curation. **Xin Cheng:** Data curation. **Liyuan Peng:** Data curation. **Yixin Tian:** Data curation. **Tiangui Li:** Data curation. **Jialing He:** Data curation. **Pengfei Hao:** Data curation. **Weelic Chong:** Data curation. **Yang Hai:** Data curation. **Chao You:** Data curation. **Fang Fang:** Data curation. **Zongjun Peng:** Conceptualization. **Yu Zhang:** Writing – review & editing, Conceptualization.

## Declaration of competing interest

The authors declare that they have no known competing financial interests or personal relationships that could have appeared to influence the work reported in this paper.

## Abbreviations

ICH: spontaneous intracerebral hemorrhage
LDH: Lactate dehydrogenase
GCS: Glasgow Coma Scale
PSI: pneumonia severity index
CURB-65: confusion, urea, respiratory rate, blood pressure, and age over 65 years
ROC: Receiver operating characteristic
AUROC: the area under the receiver operating characteristic curve
