# Supplementary material for: Elevated lactate dehydrogenase predicts pneumonia in spontaneous intracerebral hemorrhage
Source: Heliyon. 2024 Feb 13;10(4):e26109. doi: 10.1016/j.heliyon.2024.e26109 (PMC10884414; doi:10.1016/j.heliyon.2024.e26109)
Supplement: Supplementary file 1 [file mmc1.docx]

## Data Supplement

##### eFigure 1. Flow Chart of Enrollment

##### eFigure 2. Admission LDH Levels According to the Pneumonia Severity Index (PSI) Risk Groups and CURB-65 Risk Groups

A: LDH levels between the Pneumonia Severity Index risk groups

B: LDH levels between the CURB-65 risk groups

LDH: Lactate dehydrogenase

##### eTable 1. Unadjusted and Adjusted Analysis for Pneumonia.

##### eTable2. The associations Between Lactate Dehydrogenase Levels and In-hospital Mortality.

##### eFigure 1. Flow chart of enrollment


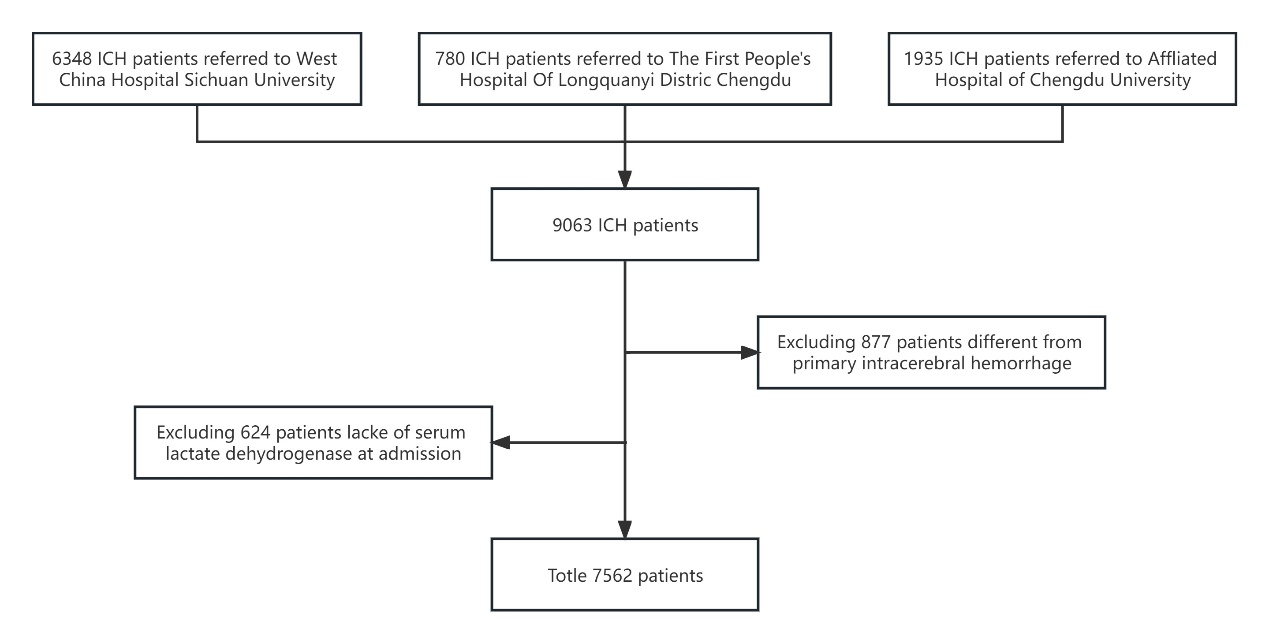


LDH: Lactate dehydrogenase

##### eFigure 2. Admission LDH levels between the Pneumonia Severity Index Risk Groups and CURB-65 Risk Groups


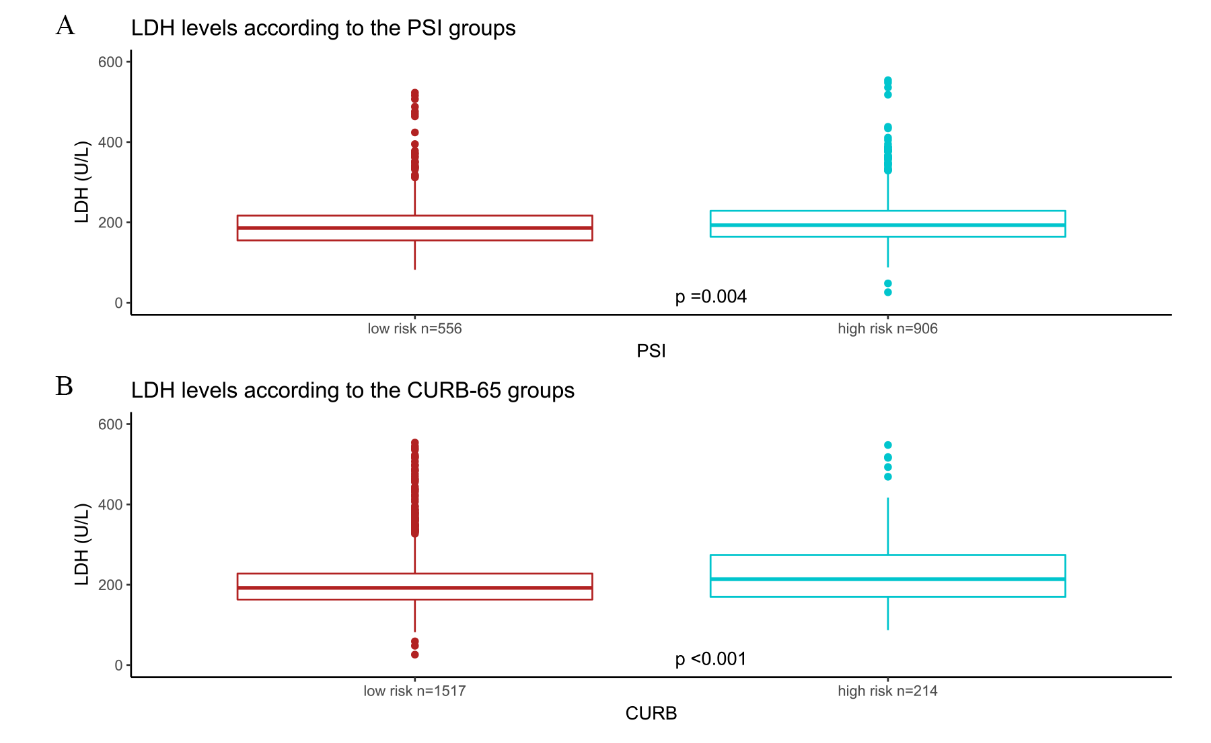


A: LDH levels between the Pneumonia Severity Index risk groups

B: LDH levels between the CURB-65 risk groups

LDH: Lactate dehydrogenase

**eTable 1. Unadjusted and Adjusted Analysis for Pneumonia.**

| Characteristics | **Unadjusted OR** | | **Multivariable Regression adjusted OR** | |
| --- | --- | --- | --- | --- |
|  | 95%CI | P value | 95%CI | P value |
| Demographics |  |  |  |  |
| Age | 1.03(1.02-1.03) | <0.001 | 1.03(1.02-1.03) | <0.001 |
| Female | 0.86(0.78-0.95) | 0.003 | 0.81(0.71-0.92) | 0.002 |
| Smoking |  |  |  |  |
| Ever | 1.23(1.11-1.36) | <0.001 | 1.15(0.99-1.35) | 0.072 |
| Current | 1.12(0.92-1.37) | 0.244 | 0.91(0.71-1.17) | 0.466 |
| Alcohol abuse | 1.22(1.11-1.35) | <0.001 | 1.11(0.96-1.29) | 0.165 |
| Medical history |  |  |  |  |
| Hypertension | 0.95(0.87-1.05) | 0.347 |  |  |
| Diabetes | 1.44(1.24-1.67) | <0.001 | 1.30(1.09-1.55) | 0.003 |
| Hematoma characteristics |  |  |  |  |
| Supratentorial hematoma | 0.93(0.83-1.05) | 0.232 |  |  |
| Size of hematoma | 1.01(1.01-1.01) | <0.001 | 1.00(1.00-1.00) | 0.054 |
| Intraventricular hematoma | 2.05(1.84-2.28) | <0.001 | 1.55(1.37-1.76) | <0.001 |
| Glasgow Coma Scale score | 0.87(0.86-0.88) | <0.001 | 0.91(0.90-0.92) | <0.001 |
| Operation |  |  |  |  |
| Hematoma clearance | 2.61(2.34-2.91) | <0.001 | 2.13(1.87-2.44) | <0.001 |
| Laboratory tests |  |  |  |  |
| Leukocyte | 1.01(1.00-1.02) | 0.02 | 1.00(1.00-1.01) | 0.249 |
| neutrocyte | 1.02(1.02-1.02) | <0.001 | 1.02(1.02-1.02) | <0.001 |
| lymphocyte | 0.63(0.58-0.68) | <0.001 | 0.85(0.77-0.93) | <0.001 |
| Albumin | 0.90(0.90-0.91) | <0.001 | 0.91(0.90-0.92) | <0.001 |
| Platelets | 1.00(1.00-1.00) | 0.047 | 1.00(1.00-1.00) | <0.001 |
| glucose | 1.01(0.99-1.02) | 0.358 |  |  |

Multivariable Regression adjusted by age, sex, smoking, alcohol abuse, diabetes, size of the hematoma, hematoma clearance operation, Size of hematoma, intraventricular hematoma, GCS, leukocyte, lymphocyte, albumin, and platelets.

LDH: Lactate dehydrogenase; GCS: Glasgow Coma Scale

**eTable2.** **The associations Between Lactate Dehydrogenase Levels and In-hospital Mortality.**

| Lactate dehydrogenase (U/L) | | Events, n (%) | Unadjusted OR | P trend | Multivariable Regression adjusted OR | P trend |
| --- | --- | --- | --- | --- | --- | --- |
| Admission | Log transformed | 595/7561(7.9%) | 50.94(30.59-84.85) | <0.001 | 7.29(4.02-13.21) | <0.001 |
|  | Q1 <163 | 72/1933(3.7%) | 1 [Reference] |  | 1 [Reference] |  |
|  | Q2 163-192 | 102/1902(5.4%) | 1.46(1.08-1.99) | 0.015 | 1.26(0.90-1.76) | 0.18 |
|  | Q3 193-231 | 137/1859(7.4%) | 2.06(1.53-2.76) | <0.001 | 1.41(1.02-1.95) | 0.04 |
|  | Q4 ≥232 | 284/1867(15.2%) | 4.64(3.55-6.06) | <0.001 | 1.90(1.40-2.58) | <0.001 |

Multivariable Regression adjusted by age, sex, smoking, supratentorial hematoma, diabetes, size of the hematoma, hematoma clearance operation, size of the hematoma, intraventricular hematoma, Glasgow Coma Scale score, leukocyte, lymphocyte, albumin, and platelets.
